# VERO® radiotherapy for low burden cancer: 789 patients with 957 lesions

**DOI:** 10.3332/ecancer.2016.677

**Published:** 2016-09-29

**Authors:** R Orecchia, A Surgo, M Muto, A Ferrari, G Piperno, MA Gerardi, S Comi, C Garibaldi, D Ciardo, A Bazani, F Golino, F Pansini, C Fodor, P Romanelli, D Maestri, V Scroffi, S Mazza, BA Jereczek-Fossa

**Affiliations:** 1 Department of Radiotherapy, European Institute of Oncology, Milan, Italy; 2 Department of Oncology and Haemato-oncology, University of Milan, Milan, Italy; 3 University of Milan, Milan, Italy; 4 Department of Medical Physics, European Institute of Oncology, Milan, Italy; 5 Unit of Radiation Research, European Institute of Oncology, Milan, Italy; * Equally contributed to the article; # Affiliation at the time of the study

**Keywords:** hypofractionation, IMRT, low burden cancer, SBRT, VERO®-system

## Abstract

**Purpose:**

The aim of this retrospective study is to evaluate patient profile, feasibility, and acute toxicity of RadioTherapy (RT) delivered by VERO® in the first 20 months of clinical activity.

**Methods:**

Inclusion criteria: 1) adult patients; 2) limited volume cancer (M0 or oligometastatic); 3) small extracranial lesions; 4) treatment between April 2012 and December 2013 and 5) written informed consent. Two techniques were employed: intensity modulated radiotherapy (IMRT) and stereotactic body radiotherapy (SBRT). Toxicity was evaluated using Radiation Therapy Oncology Group/European Organisation for Research and Treatment of Cancer (RTOG/EORTC) criteria.

**Results:**

Between April 2012 and December 2013, 789 consecutive patients (957 lesions) were treated. In 84% of them one lesion was treated and in 16% more than one lesion were treated synchronously/metachronously; first radiotherapy course in 85%, re-irradiation in 13%, and boost in 2% of cases. The treated region included pelvis 46%, thorax 38%, upper abdomen 15%, and neck 1%. Radiotherapy schedules included <5 and >5 fractions in 75% and 25% respectively. All patients completed the planned treatment and an acceptable acute toxicity was observed.

**Conclusions:**

RT delivered by VERO® was administrated predominantly to thoracic and pelvic lesions (lung and urologic tumours) using hypofractionation. It is a feasible approach for limited burden cancer offering short and well accepted treatment with favourable acute toxicity profile. Further investigation including dose escalation and other available VERO® functionalities such as real-time dynamic tumour tracking is warranted in order to fully evaluate this innovative radiotherapy system.

## Introduction

In recent years new radiotherapy modalities have been widely introduced allowing for high precision dose delivery and verification. Numerous systems are commercially available. One of these is the VERO® system (Mitsubishi Heavy Industries, Ltd., Tokyo, Japan and Brainlab AG, Feldkirchen, Germany) which is a dedicated machine for image-guided SBRT.

The VERO® system is equipped with a ring that is capable of rotating ± 60° about the vertical axis to facilitate non-coplanar beam arrangements ideal for stereotactic ablative body radiotherapy (SABR) delivery. The beam delivery platform consists of a 6 MV C-band linac with a 60 leaves multileaf collimator(MLC) projecting a maximum field size of 15 × 15 cm^2^ at the isocentre [1]. The planning and delivery systems offer a combination of static conformal beams, dynamic conformal arcs, fixed gantry intensity modulated radiotherapy (IMRT) with either static MLC (step-and-shoot) or dynamic MLC (DMLC), and Hybrid-Arcs, which combine dynamic conformal arcs and fixed beam IMRT delivery. Moreover, the linac is mounted on two orthogonal gimbals (robotic arcs) allowing pan and tilt rotation of the beam to perform dynamic tumour tracking.

The imaging system consists of two orthogonal x-ray tubes attached to the O-ring at ± 45° from the MV beam axis and operating at potentials of 40–150kV. Image guidance can be performed in different ways: using the two kV systems simultaneously in a radiographic or in the fluoroscopic mode with a frequency of about 15 frames/sec or using a single tube to acquire a cone-beam computer tomography (CBCT)with a maximum field of view (FOV) diameter of 20 x 15 cm. The VERO® system is also equipped with an amorphous silicon electronic portal imaging detector (EPID) permanently mounted in the ring [1]. Finally, an ExacTrac (Brainlab AG, Feldkirchen, Germany) automated infra-red marker based patient-positioning device and a 5 degrees-of-freedom robotic couch are also integrated into the system.

Radiotherapy (RT) delivered by VERO® was implemented in our department in April 2012, and it was one of the first installations worldwide. In 2012 we started using all mentioned VERO® system performances in our clinical practice with the exception of real-time tumour tracking. At present, the implementation of this feature is in-progress [2]. Based on the technical characteristics of the system, the general indications for RT delivered by VERO® included low burden cancer patients: organ confined-prostate cancer, prostatic bed, and extra-cranial small volume primary or oligometastatic tumours [3–5].

The aim of this retrospective study is to evaluate the patient profile, feasibility, and acute toxicity of RT delivered by VERO® in the first 20 months of clinical activity.

## Materials and Methods

### Study protocol

This is a retrospective study on all consecutive patients treated with RT delivered by VERO® in the first 20 months of its activity. The study was a part of general SBRT and image-guided radiation therapy (IGRT) research notified to the Ethical Committee of the European Institute of Oncology, Milan, Italy (notifications No. 79/10, 86/11, 87/11, 93/11). All patients gave written consent for the use of their completely anonymised clinical and imaging data for educational and research purposes.

### Inclusion criteria

The inclusion criteria for the RT delivered by VERO® in our department were as follows: 1) more than 18 years old; 2) limited volume cancer (M0 or oligometastatic), i.e. favourable prognosis (because of high complexity of treatment); 3) small extracranial lesions (because of geometry of VERO® beam); 4) treatment performed between April 2012 and December 2013 and 5) written informed consent. Previous radiotherapy or concomitant systemic therapy were allowed.

The cancer extent was based on clinical examination and imaging studies including bone scan, computer tomography (CT), magnetic resonance imaging (MRI), [^18^F]fluoro-deoxy-glucose positron emission tomography/CT scan ([^18^F]FDG-PET/CT), or in case of prostate cancer, Choline C-11 positron emission tomography/CT scan ([^11^C]choline-PET/CT). The disease localisation was divided according to the following categories: primary tumour (T), regional lymph node (LN), or distant metastasis (M).

In admitting patients to the study, any kind of previous or concomitant cancer therapy was permissible. In case of overlapping with previous radiotherapy fields, the original plan data was required. Those patients who had begun systemic treatment before or concomitantly with radiotherapy were also admitted. The treatment was performed either with curative intent (single cancer lesion or oligometastatic disease) or palliative extent (small symptomatic lesions with favourable histology, etc.).

### Treatment protocol

All patients were immobilised during both CT scanning and treatment, using a customised external vacuum-type cast. Image fusion of CT with MRI was applied for some cases (mainly prostate cancer) to guide contouring of the target and organs at risk. No radio-opaque fiducial marker was introduced into any target lesion.

CT simulation (2.5 mm slice thickness) was performed on a 4-slice clinical scanner (Lightspeed, GE Healthcare, Milwaukee, WI). Seven passive markers were placed on stable points of the patient chest or abdomen for patient positioning with ExacTrac during every fraction. No routine contrast medium was used for simulation CT (it was used for small lesions like lymph nodes or for liver lesions).

Institutional guidelines were used for the contouring of the tumour and organs at risk. A 3–8mm margin was added to the gross tumour volume (GTV) in order to create the planning target volume (PTV). In case of thoracic lesions the GTV-PTV margins was 3–8 mm; for abdominopelvic lesions the GTV-PTV margin was 3–5 mm.

**Figure 1.**
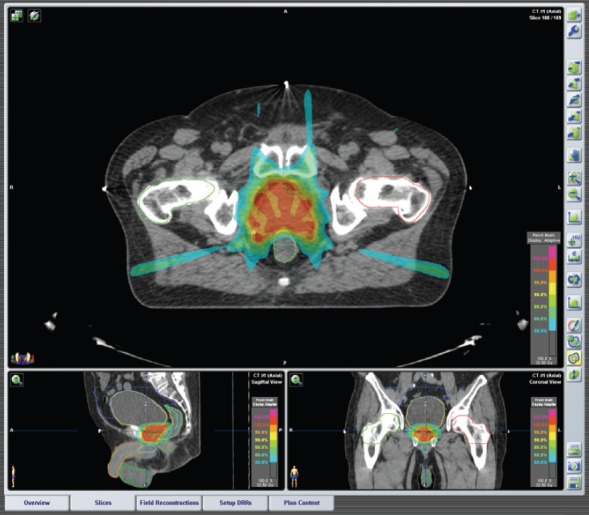
Plan of IMRT Prostate cancer, stage: cT1c cN0 cM0. IMRT treatment plan: Seven fixed beams, Energy: 6MV XRays. Total dose: 32.5 Gy in five fractions (6.5 Gy/fraction) every other day.

**Figure 2.**
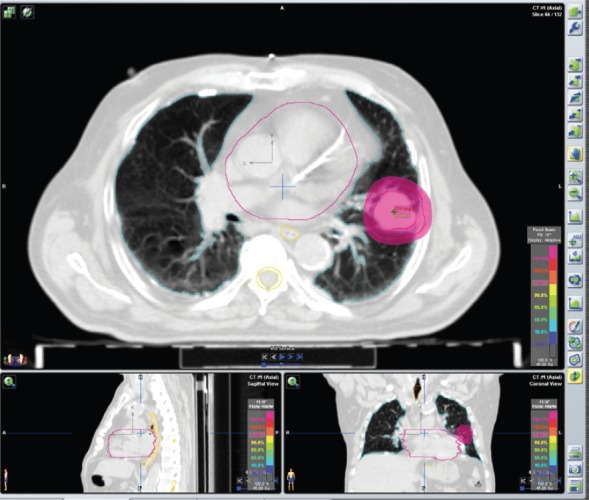
Plan of SBRT Left lung cancer, stage: cT2 cN0 cM0. SBRT treatment plan: Two dynamic arcs, Energy: 6MV XRays. Total dose: 45 Gy in three fractions (15 Gy/fraction) every other day.

iPlanRT(v 4.5, Brainlab, Feldkirchen, Germany) was used to elaborate the plans. Two techniques were employed: IMRT and SBRT. Step and shoot IMRT technique was applied using fixed gantry approach with 5–7 fields in order to guarantee an optimal PTV coverage (Figure 1). Dynamic arcs, 1–3 coplanar or non-coplanar were usually used for SBRT treatments (Figure 2), although conformal beams or IMRT were also applied in selective cases. We can obtain non-coplanar fields moving the ring of ±30° around the vertical axis (the maximum rotation of ±60° is hardly achievable in the clinical practice). High dose per fraction (>5 Gy/fraction), few treatment fractions, small treatment volumes, IGRT, are common criteria of SBRT.

The choice of IMRT or SBRT depended on the PTV volume and shape, and its position with respect to the organs at risk (OARs). The prescribed doses depended on the tumour histology and cancer extent. In general, hypo-fractionated regimens were applied with long schedule (>5 fractions) or short schedule( ≤5 fractions, i.e. ultra-hypofractionation).

In cases of short schedules the dose was up to 20 Gy per fraction and in cases of long schedules it was up to 8 Gy per fraction. The prescription could vary with higher dose per fraction in case of single cancer localisation or lower dose in case of re-irradiation or proximity to critically radiosensitive structures, such as intestinal loops. The dose volume constraints for OARs reported by Timmerman *et al* were respected [6]. In case of re-irradiation, the constraints were lowered based on the previous radiotherapy plan data [7].

In cases of long schedule, used mainly for prostate cancer or partial breast irradiation treatments, regimens between 1.8 Gy and 2.85 Gy per fraction were applied according to the institutional policy (curative or post-prostatectomy prostate cancer radiotherapy, adjuvant breast cancer radiotherapy). The doses were delivered on the consecutive or alternating days in case of long schedule and short schedule respectively.

Patients were positioned using ExacTrac and the target was localised by means of a 200° rotation CBCT. The x-ray tube parameters were optimised to take account of the patient’s characteristics in order to obtain a good image quality. For the lung, our standard lung protocol was 110 kV, 100 mA, 5 ms, while for the pelvis was 110 kV, 250–320 mA, 8 ms. A preliminary match between CBCT and simulation CT scans was obtained by an automatic image registration and then manually refined by a radiation oncologist for improved target alignment (manual soft tissue registration). Detected misalignments in terms of translations and rotations were corrected by the robotic couch motion and O-ring motion in order to minimise deviations with respect to patient position at simulation CT.

### Patient monitoring

All treatments were performed on an out-patient basis. Steroid premedication was administrated in case of short schedules and depended on the clinical scenario (symptoms, tumour location, etc.).

In cases of short schedules, the patients were seen by the radiation oncologist before and after each fraction. The patients undergoing a long schedule were seen once a week.

The criteria of the Radiation Therapy Oncology Group/European Organisation for Research and Treatment of Cancer (RTOG/EORTC) were used to evaluate treatment toxicity [8]. Acute toxicity was analysed in all patients (the degree of toxicity occurring during and at the end of radiotherapy treatment).

After the treatment, check-up visits were scheduled every 2–4 months (and every 6–12 months in case of prostate cancer patients). Prostate cancer patients were monitored with prostate serum antigen (PSA), radiological evaluation was requested only in case of PSA progression or for clinical suspects. In other patients routine radiological or [^18^F]FDG-PET/CT re-evaluation was performed. All patient data collected for this study were archived in the institutional database.

### Statistical analysis

The primary aim of this study was the description of patient profile, feasibility, and acute toxicity of the treatment. Late toxicity and tumour control of RT delivered by VERO® will be a subject of the future study.

Patient characteristics were represented as frequencies and percentages for categorical variables, and medians and ranges for continuous variables. The information on patient’s tumour, and treatment related data were registered.

## Results

### Patients and treatment

Between April 2012 and December 2013, 789 consecutive patients were treated with 964 lesions. In 660 patients (84%), one lesion was treated whereas in 129 patients (16%) more than one lesion were treated synchronously/metachronously. Median age was 70 years (range, 20.3–91.4 years) and male/female ratio was 541(69%)/248(31%) (Table 1).

RT delivered by VERO® was a first radiotherapy course for 813 patients (85%), re-irradiation for 127 patients (13%), and boost for 17 patients (2%). Median interval between diagnosis of primary tumour diagnosis and RT was 19.8 months (range, 0.6–299.4 months).

Primary diagnosis included urologic tumours (354 patients, 45%), lung tumours (176 patients, 22%), gastrointestinal tumours (94 patients, 12%), breast tumours (72 patients, 9%), gynaecological tumours (60 patients, 8%), head/neck tumours (8 patients, 1%) and other malignancies (melanoma, thymoma, unknown primary tumour, etc.) (25 patients,3%).

The treated region included, pelvis (441 lesions, 46%), thorax (363 lesions, 38%), upper abdomen (140 lesions, 15%), neck (13 lesions, 1%). T, N, and M lesions were treated in 476 (50%), 139 (14%), and 342 (36%) patients respectively. M category included bone, nodal, and visceral metastases.

The treatment schedules included <5 and >5 fractions (short and long schedule) in 75% and 25%, respectively.

Median total dose was 29 Gy (range, 5–76). In the short schedules the median total dose was 25 Gy (range, 5–60 Gy), and in the long schedules the median total dose was 42.1 Gy (range, 20–76 Gy).The median number of fractions was 4 (range, 1–5) and 20 (range, 6–38) in the short and long schedules respectively. Median dose per fraction was 7.5 Gy (range, 1.8–20 Gy) and 2.8 Gy (range, 1.8–8.0 Gy) in the short and long schedules respectively. Median overall treatment duration was 11 days (range, 1–54 days). Mean duration of single fraction SBRT and IMRT (with CBCT evaluation) was 16 minutes.

In 261 cases (27% of lesions) concomitant systemic therapy was performed: chemotherapy only, hormonal only, and both treatments in 22%, 75%, and 3%, respectively.

All patients completed planned treatment. The treatment was well tolerated where 69% of patients did not experience any toxicity. A total of 247 patients (31%) experienced acute toxicity during or after treatment. Severe acute toxicity RTOG/EORTC grade ≥3 occurred rarely (Table 2). These events included six events of grade 3 and 2 events of grade 4 genitourinary toxicity (two cases of acute urinary obstruction), one event of grade 3 pulmonary toxicity. Data were analysed for the occurrence of severe acute toxicity and the course of radiotherapy (first versus re-irradiation): only one event of grade 3 dyspnoea occurred in re-treated pulmonary lesion. All cases of genitourinary grade 3 and 4 toxicity occurred during first irradiation of prostatic tumours. Nevertheless, all severe events were temporary as noted that at the first follow-up visit (three or six months after radiotherapy) all patients were asymptomatic.

## Discussion

Our analysis has demonstrated a feasibility and very good toxicity profile of VERO® irradiation including IMRT and SBRT treatments. The current study is a part of our experience in high precision selective radiotherapy technologies allowing for non-toxic hypofractionation for limited volume primary and metastatic cancer [7, 9–10].

To the best of our knowledge this is the first largest clinical series on radiotherapy using the VERO® system. The other available reports at present concern either the technical or dosimetric issue of the VERO® system [1, 11] or its limited clinical applications [12].

Our study included 789 consecutive patients (957 lesions) treated in one institution with VERO® over just the first 20 months. Altogether 2600 patients were treated with this modality at our department until the 30 June 2016. The whole patient population and the follow-up data will be the subject of future study. In the current series, the side effects were generally mild and the majority of patients did not experience any toxicity.

Because of VERO®’s versatility, the treatment of any small target in any area of the body was feasible. The median interval between diagnosis of primary tumour and RT of 19.8 months shows that both primary tumours and recurrent or metastatic lesions were included.

In our experience RT delivered by VERO® was administrated predominantly to pelvic and thoracic lesions (lung and urologic tumours) using hypofractionated schedules. The patient’s case profile with small tumour volumes corresponds well with the geometry of machine, i.e. small treatment fields [1].

Excellent target coverage and low doses to the surrounding normal tissue allow the employment of RT delivered by VERO® as a curative ablative therapy or re-irradiation in many patients. The high quality CBCT allows for comfortable target positioning before each radiotherapy session. Relatively short treatment time guarantees limited intra-fraction organ motion. Therefore, in our daily practice we did not use implantable fiducials and base image guidance on the CBCT. This decision has been made in order to reduce the invasiveness of radiotherapy procedures and increase patient’s compliance. Although only up to 8% of infectious complications and 1% of sepsis have been reported in the literature after the fiducial positioning [13–14], this risk has risen over the last years because of antibiotic resistance and increases in patient’s comorbidities (the median age of our patients is 70 years) [13–14]. Complications after material positioning in the retroprostatic area have also been published [15]. However, the issue of the optimal image-guidance continues to be controversial [16]. Importantly, daily CBCT seems to be an acceptable and reliable way to check patient’s target positioning if performed by trained staff [17–18].

In our series ultra-hypofractionated treatment was always performed on alternating days as we have seen a correlation of it with reduction in toxicity [19]. Currently, we are implementing the real-time tumour tracking (RTTT) system for treatment of moving tumours (especially for lung or liver cancer) since our department is equipped with 4-dimensional CT (4D CT) [2].

It is well known that IMRT can improve dose distributions and reduce radiation doses to adjacent normal tissues with appropriate planning. Hence for our study this was the criteria for the selection of patients, i.e. those who have had IMRT for pelvic and thoracic tumours performed (mainly breast and lung tumours). It is the same advantage with primary thoracic and abdominopelvic tumours treated with SBRT [20–23]. The choice between SBRT and IMRT is based on the tumour shape and localisation.

The selection criteria for SBRT in treating oligometastatic cancer remain unclear [22, 24–26]. In several reports oligometastatic condition suitable for SBRT is defined by the limited number of metastases (up to five), a limited tumour diameter, and a locally controlled primary tumour. Other authors recently proposed selection criteria based on the favourable histology(germ cell tumours, non epithelial cancer, etc.), limited metastatic disease, the metachronous appearance of metastases, young age, and a good performance status of the patient [22, 24–26].

We already had a strong experience in SBRT treatment for oligometastatic patients with lymph node recurrent cancer. It was excellent in field tumour control and yielded very low toxicity profile [10, 27–30]. For this experiment, we selected the oligometastatic patients who were not candidates for surgery or other invasive therapies. The selection was also based upon comorbidities, age, performance status, and patients in whom it was important to minimise the toxicity, the duration of local treatments, or to postpone systemic therapies (treatment-free interval). These objectives were met in the majority of our patients and this makes RT delivered by VERO® an attractive treatment modality especially for strongly pre-treated patients.

Acute toxicity was limited(only nine cases of ≥G3 acute toxicity, i.e.1.1% of all patients). The evaluation of late toxicity and tumour outcome requires longer follow-up and will be the subject of the future report. Based on the favourable preliminary experience with RT delivered by VERO®, both from the patient and health professionals points of view, the enrollment for this highly selective local treatment modality continues, as confirmed by the numbers mentioned above (about 2600 patients already treated).

We are well aware of other limitations in our study, like the lack of late toxicity and tumour control data, heterogeneity of the patients in terms of treatment site (all the body districts were analysed) as well as in terms of primary tumours histology, volume, and stage, previous and concomitant therapies, treatment intent (curative and palliative treatments), radiotherapy schedules, and modalities (IMRT versus SBRT). T,N, and M lesions at first and/or with re-treatments were analysed together without discriminations. Some interpretation problems can also result from the fact that in M category bone, visceral, soft tissue lesions, and lymph node metastases were included. However, the purpose of our report was to present the first hand experience with VERO® system used for IMRT and SBRT.

## Conclusion

In conclusion, RT delivered by VERO® is a feasible approach for limited cancer offering a short and well accepted treatment with extremely favourable acute toxicity profile. Further investigation is warranted to evaluate tumour control and late toxicity in specific patient subgroups (by tumour site, treatment intent, etc.) and to evaluate all potentials of the VERO® system (real time tumour tracking, dose escalation, etc.).

## Conflict of Interest

None.

## Figures and Tables

**Table 1.** Patient, tumour, and treatment characteristics (N = 789 patients, n = 957 lesions).

Characteristics	All patients N = 789
Age (years), at the treatment Mean Median Range	68 70 20.3–91.4
Gender Male Female	541 (69%) 248 (31%)
Primary diagnosis Urologic (prostate) tumours Lung tumour Gastrointestinal tumours Breast tumours Gynaecological tumours Urologic (non-prostate) tumours Head/neck tumours Other primaries	338 (43%) 176 (22%) 94 (12%) 72 (9%) 60 (8%) 16 (2%) 8 (1%) 25 (3%)
Interval between diagnosis of primary tumour and RT/per lesion (n = 957) Median range in months	19.8 0.6–299.4
Number of treated lesions per patient 1 2 3 4 5	660 (84%) 101* (13%) 21^ (2%) 3° (0.4%) 4^§^ (0.6%)
Treated region per lesion (n = 957) Pelvis Thorax Upper abdomen Neck	441 (46%) 363 (38%) 140 (15%) 13 (1%)
Treatment group per lesion (n = 957) T N M	476 (50%) 139 (14%) 342 (36%)
Treatment course per lesion (n = 957) 1° treatment re-irradiation boost	813 (85%) 127 (13%) 17 (2%)
Concomitant systemic therapy per lesion (n = 957) No Yes Chemotherapy Endocrine therapy Both	696 (73%) 261 (27%) 56 (22%) 197 (75%) 8 (3%)
Hypofractionation per lesion (n = 957) Short schedule (< 5 fractions), i.e. ultra-hypofractionation) Long schedule (> 5 fractions)	713 (75%) 244 (25%)
Total dose (Gy) in the short schedules (< 5 fractions) Median range Mean	25 5–60 31.5
Total dose (Gy) in the long schedules (> 5 fractions) Median range Mean	42.1 20–76 53.1
Dose (Gy) per fraction in the short schedules (< 5 fractions) Median range Mean	7.5 1.8–20 7.8
Dose (Gy) per fraction in the long schedules (> 5 fractions) Median range Mean	2.8 1.8–8.0 2.9
Fractions number in short schedules (< 5 fractions) Median range Mean	4 1–5 4.3
Fractions number in long schedules (> 5 fractions) Median range Mean	20 6–38 20.1

Legend: RT – radiotherapy, T – tumour, N – regional lymph node, M – metastases

* Two lesions were treated metachronously in seven cases (1 + 1)

^ Three lesions were treated metachronously in three cases (1 + 1 + 1)

° Four lesions were treated metachronously in two cases (2 + 2 e 3 + 1)

§ Five lesions were treated metachronously in one case (4 + 1)

**Table 2.** Acute treatment toxicity according to the criteria of the Radiation Therapy Oncology Group/European Organisation for Research and Treatment of Cancer (RTOG/EORTC) [8].

Treated region	Acute toxicity	Number of events N = 957 lesions
Pelvis 441 lesions	**GU** G1 G2 G3 G4 Total	112 (25%) 34 (8%) 6 (1.4%) 2 (0.5%) 154 (35%)
	**GI** G1 G2 G3 G4 Total	71 (16%) 14 (3%) 0 0 85 (19%)
Thorax 363 lesions	**Lung toxicity** G1 G2 G3 G4 Total	23 (6%) 4 (1%) 1 (0.3%) 0 28 (7.3%)
Thorax + Neck 376 lesions	**Skin toxicity** G1 G2 G3 G4 Total	27 (7%) 4 (1%) 0 0 31 (8%)

The degree of toxicity was evaluated during and at the end of treatment (maximum grade observed)

Legend: lesions = total number of treated lesions per region, GU = genitourinary toxicity, GI = gastrointestinal toxicity. G = grade, RTOG/EORTC = Radiation Therapy Oncology Group/European Organisation for Research and Treatment of Cancer
